# Subcategory vs category fluency: Items and networks in healthy young adults and simulation with a large language model

**DOI:** 10.3758/s13421-026-01869-3

**Published:** 2026-04-08

**Authors:** Adrià Rofes, Demi van Dijk, Jeffrey C. Zemla

**Affiliations:** 1 Center for Language and Cognition Groningen (CLCG), Faculty of Arts—Neurolinguistics and Language Development, University of Groningen, Oude Kijk in ‘t Jatstraat 26, 9712EK Groningen, The Netherlands; 2 Research School of Behavioural and Cognitive Neurosciences, University of Groningen, Groningen, The Netherlands; 3 Koninklijke Kentalis, Sint-Michielsgestel, The Netherlands; 4 Department of Psychology, Syracuse University, Syracuse, NY USA

**Keywords:** Category, Subcategory, Fluency, Word properties, Clusters, Networks, LLM

## Abstract

Category fluency tasks involve producing words constrained by a semantic field (*animals*). Subcategory fluency involves producing words from categories that are semantically related to a superordinate category but form a restricted set of items (*farm animals*). Here, we study whether people produce different patterns of words in category versus subcategory fluency by looking at differences in the total number of words produced, the properties of the words produced (e.g., frequency) and how people group words together (clusters/switches and network metrics). Forty-eight Dutch-speaking university students responded to three category fluency tasks (*animals, foods, transport*) and three subcategory fluency tasks (*farm animals, fruits, bike parts*). Also, we queried a large language model (LLM) to provide responses for 50 “pseudo-participants” for the same six categories. People in category (versus subcategory) tasks produced more words; words of higher frequency, with fewer orthographic and phonological neighbors, and shorter in length. They also produced fewer cluster switches and bigger clusters. The category and subcategory networks had different structure (e.g., number of nodes, edges, clustering coefficient). With the LLM we simulated the results regarding word properties and cluster size, but found differences regarding correct words, number of switches, and overlapping clusters between *foods* and *fruit* fluency. The differences between category and subcategory fluency may stem from differences in mental search in the lexico-semantic system. However, category and subcategory fluency tasks may be different tasks altogether. The LLM simulation provides novel insights (e.g., how words relate, task-order effects) and suggests caution when used to understand human fluency data.

**Supplementary Information:**

The online version contains supplementary material available at 10.3758/s13421-026-01869-3.

## Introduction

Fluency tasks are widely used in research and clinical practice (Rofes et al., 2017, 2022; Rook et al., 2023). Individuals are typically given 1 minute to name as many unique words as possible (De Marco et al., 2023). The two most common types of fluency tasks are category fluency and letter fluency. Category fluency (or semantic fluency) requires the production of words that belong to a specific category (e.g., *animals, foods, fruits*). In letter fluency (or phonological fluency), words must begin with a specific letter of the alphabet (e.g., *F, A, S*). Fluency tasks tap into linguistic abilities and executive functioning. This argument is supported, among other findings, by correlations of total number of correct words and measures such as vocabulary tests and operation span tasks (Shao et al., 2014; Whiteside et al., 2016), and with studies relating total number of correct words with item-based measures, such as clusters/switches and word properties (De Marco et al., 2023; Rofes et al., 2023). In this paper, we investigate fluency of general (or superordinate) categories (e.g., *animals, foods, transport*) and fluency of “subcategories” (e.g., *farm animals, fruits, bike parts*). This work is novel given that subcategory fluency tasks are not often administered or compared with category fluency tasks. Afterward, we conduct identical analyses on fluency data generated by an open-source transformer-based large language model (LLM) tuned for Dutch (i.e., Mistral 24B). Our results show some consistencies across human and LLM data, suggesting that LLM models may provide novel insights (but suggest caution) to study fluency tasks.

### A definition of subcategory fluency

In a subcategory fluency task participants are asked to produce words belonging to a specific semantic category, such as farm animals, fruits, and bike parts. Subcategory fluency implies that the words produced are semantically related and represent a more restricted set of items than the general or superordinate category. For example, *farm animals* and *fruits* are semantically related, represent a more restricted set of items, and are a subset of *animals* and *foods*, respectively. In other words, items like “sheep” and “banana” are farm animals and fruits, but they are also animals and foods, respectively. In this research, we also define subcategory fluency to include producing words that are part of a specific object of the general category. For example, *bike parts* are semantically related and represent a more restricted set of items than means of *transport.* However, *bike parts* are not a subset of the category *transport* (i.e., they are not nested). To give an example, items like “handlebar” or “saddle” are semantically related to a “bicycle,” which is a means of transport. However, neither “handlebar” nor “saddle” is a means of transport.

### Do category and subcategory fluency have similar lexico-semantic patterns?

A theoretical motivation behind this study is to explore whether there are differences between category and subcategory fluency, and whether these differences reflect distinct search strategies or mental representations. One possibility is that any differences are directly influenced by the size of the available word pool, in which case similar results are expected regardless of the semantic field to which the words belong to (e.g., *animals*, *transport*, *foods*). Alternatively, differences could depend on the semantic field and/or other characteristics of the words (e.g., frequency, length in graphemes, phonological/orthographic neighbors). In this second case, differences between category fluency and subcategory fluency might emerge for some semantic fields, but not all.

To answer to this question, we will ask whether the lexico-semantic search to produce words during category fluency tasks differs when context is constrained (superordinate category versus subcategory). One possibility is that word production during category fluency is driven in part by automatic associations between similar responses (Marko et al., 2023; Rende et al., 2002; Troyer & Moscovitch, 2012)—but what counts as similar? The work of Goldstone et al. (1997) and Tversky (1977) becomes relevant, particularly, as these authors indicated that in similarity judgment tasks participants group items differently based on the surrounding context. For example, participants in Tversky (1977) were asked to select the country most similar to Austria: Sweden, Hungary, or a third option (either Norway or Poland). When the third option was Poland, participants viewed Austria as more similar to Sweden than Hungary; when the third option was Norway, participants viewed Austria as more similar to Hungary than Sweden.

Going back to the discussion of context and fluency tasks, in *animal fluency* data people often group animals that are similar in “habitat” (e.g., farm animals, pets, aquatic, Australia) or taxonomy (e.g., birds, reptiles). This sense of similarity is made explicit in hand-coded schemes. For example, Troyer (2000) discusses different types of *clusters*—that is, collections of words consecutively produced during fluency tasks that share specific features. Some of the clusters that can commonly appear in fluency tasks are for words like farm animals (e.g., chicken, cow, donkey), pets (e.g., canary, cat, dog), and birds (e.g., condor, eagle, finch). However, animals can be similar in other ways, such as size, color, or the products we obtain from them (e.g., meat, fur). In the case of a subcategory of animals, such as *farm animals*, the option of grouping by habitat disappears or, at least, it is less obvious than for the general category of animals (e.g., crops, pastures, barns, vineyards). As a matter of fact, given that the goal of a farm is “growing crops and rearing animals”, in the context of a farm, animals may be better grouped by their purpose to humans (e.g., dairy and meat, work, fur and fiber). A question we can ask, therefore, is whether the dominant association(s) used to guide search in *animal fluency* are similar to the associations used to guide search in *farm animal fluency*? In other words, if we change the context from “animals” to “farm animals,” does that change how people engage in lexico-semantic search?

We explore this latter question by examining how people transition between farm animals within *animal fluency*, and compare that to how people transition between farm animals in *farm animal fluency*. If people rely on the same strategy or have a common representation for both category and subcategory fluency, they may produce similar clusters of farm animals in *farm animal fluency* and *animal fluency*. However, if they use different strategies or representations, the clusters produced will likely differ between these tasks. For example, in *farm animal fluency*, people might cluster responses by purpose (e.g., dairy and meat), while clusters of farm animals within *animal fluency* might not have an organizational structure as fine-grained.

### Are effects of context equally present between fluency tasks with different semantic fields?

Another question we will ask is whether effects of context are equally present when participants respond to different types of category fluency tasks. In other words, does context affect the lexical search of “animals vs farm animals,” “foods vs fruits” in the same way? And how about subcategories that do not represent a subset of items but are semantically related, like “bike parts” from “transport”? This question stems from dissociations reported in people with language impairments after brain damage. For example, Hillis and Caramazza (1991) described the data of two individuals with brain damage due to stroke in the left temporal lobe. One showed no impairments for the production and comprehension of animals, but did show impairments in other categories, such as vegetables, fruits, foods, or clothing. Another individual showed the opposite pattern. People with brain infections due to herpes simplex encephalitis have further difficulties with biological entities (e.g., deer, grapefruit) than artifacts (e.g., hammer, guitar) in a number of tasks, including naming and picture description (Warrington & Shallice, 1984; for a review Rofes et al., 2022). Specific to fluency tasks, Ventura et al. (2005) asked undergraduates to generate as many words as possible (biological and artifacts) given four sensory criteria (i.e., small, big, light, heavy) and four functional criteria (many people own, fewer people own, people own for pleasure, people own for necessity). The authors indicated that fluency tasks with sensory criteria elicited more biological items and clusters of biological items, while fluency tasks with functional criteria elicited more artifacts and clusters of artifacts. The data of Ventura et al. (2005) is in line with the findings of Warrington and Shallice (1984), as it indicates that effects of context in fluency tasks may affect the types of lexical items produced. In our view, this can reflect differences in how the lexical search is conducted, for example, how participants associate words in each of the tasks, given that in subcategory tasks the possible set of words to be produced is more limited than in category fluency task.

### Previous subcategory fluency studies: focus on total number of correct words

Studies discussing the types of subcategories that participants use in a general fluency task, as well as studies administering up to 70 different types of category fluency tasks exist (Arroyo-Anlló et al., 2011; Moreno-Martínez et al., 2016; Price et al., 2012; Van Overschelde et al., 2004). However, to the best of our knowledge, differences regarding the items produced and how the items group together specifically between superordinate category and subcategory fluencies, have not been systematically investigated. Most work looking at subcategory fluency has analyzed the total number of correct words between tasks and/or participants. Arroyo-Anlló et al. (2011) showed that individuals with Alzheimer’s disease and people with fluent poststroke aphasia name significantly fewer words than healthy individuals during *supermarket fluency*. Interestingly, the authors argued that people with Alzheimer’s disease have difficulties at the semantic level because, compared with the other groups, they generated more generics (e.g., fruits, fish, vegetables), and a reduced proportion of specific exemplars (e.g., banana, sardine, lettuce) within a subcategory (e.g., grocery items). Moreno-Martínez et al. (2016) investigated 14 different fluency tasks, including two subcategories of *foods fluency*: *fruit fluency* and *vegetable fluency*. Moreno-Martínez et al. (2016) showed that these subcategory tasks have a high value for sensitivity and low value for specificity to detect/reject people from Alzheimer’s disease from healthy controls. The authors provided low values for sensitivity and specificity for *animal fluency*, which is probably one of the most commonly used category fluency tasks (De Marco et al., 2023; Rofes et al., 2017, 2022; Rook et al., 2023).

### Other methods to study category and subcategory fluency

There exist many other methods to study fluency tasks. To the best of our knowledge, many of these methods have not been explored in the context of comparisons between category versus subcategory fluency tasks:

The first method we will look at is studying how items group together in terms of *clusters and switches*. Clusters represent collections of words that are semantically similar (Troyer, 2000) and a measure that can be obtained from the analysis of clusters is *cluster size*. That is, the number of consecutive responses that belong to the same cluster. For example, the series “hamster, cat, dog” forming a cluster of size three. The mean cluster size of the same fluency task across different participants can be calculated, taking the name of *average cluster size.* Another measure that can be obtained is the number of *cluster switches*. That is, how many times the participant moves from one cluster to another. For example, in the sequence “*chicken, cow, donkey, condor, eagle, finch*” there is one *cluster switch* between the farm animal cluster “chicken, cow, donkey,” and the bird cluster “*condor, eagle, finch.*”

Many researchers have looked at average cluster size and number of cluster switches in healthy and clinical populations. For example, Price et al. (2012) compared *animal* and *supermarket fluency* in people with amnestic mild cognitive impairment (aMCI). In contrast to healthy controls, people with aMCI produced fewer words, fewer novel subcategories, and smaller clusters in these tasks. Differences in clustering and switching behavior can also vary by category (e.g., Rosselli et al., 2009). Many clinical populations are known to have deficits in clustering and/or switching. For example, Alzheimer’s patients generate smaller clusters and switch clusters less frequently than healthy controls (Troyer et al., 1998). There is also variance among healthy populations—for example, adults switch clusters less frequently with age (Zemla et al., 2020). Impairments in clustering and switching behavior are often thought to reflect deficits in associative representations and in executive functioning, respectively (Mayr, 2002; Troyer et al., 1997).

A second method is to study the *properties of the words* (i.e., word properties or psycholinguistic variables) produced in the fluency tasks. McClelland and Rogers (2003) described that words in the same subcategory are closely related. When one word in the subcategory is retrieved from the lexicon, it is easier to retrieve other words from the same subcategory. Vonk et al. (2019) indicated that people at risk of Alzheimer’s disease produced words with higher frequency during animal fluency compared with people that were not at risk of Alzheimer’s disease. Rofes et al. (2019) indicated that familiarity, a word property that is typically affected in people with semantic impairments could be used to differentiate individuals with semantic variant primary progressive aphasia compared with people with two other primary progressive aphasia variants (i.e., nonfluent, logopenic). Rofes et al. (2021) argued that individuals with Human Immunodeficiency Virus may have difficulties at the lexico-semantic level, provided that their total number of words in fluency tasks could be predicted with word frequency.

Other word properties that can be studied are phonological and orthographic neighborhood density (Marian et al., 2012; i.e., the number of words that can be formed from a single phoneme or grapheme substitution). For example, in English, the phonological neighborhood density for a word like “eagle” is nine as given the phonological form of the word (i.e.,/i.g.l/) there are nine other words differing from/i.g.l/in one phoneme by either substitution, deletion, or addition (e.g., ogle, eel, Beagle). Using the same method, but now considering graphemes, the orthographic neighborhood density of eagle is three (i.e., Engle, Eagles, Beagle). Importantly, effects of neighborhood density have been related to lexical processing (Whitworth et al., 2014). For example, healthy participants are slower when naming objects with high phonological neighborhood density (Sadat et al., 2014) or reading words with high orthographic neighborhood density (Perea & Rosa Martínez, 2000). Finally, the length of the word measured as the number of graphemes can also affect the total number of words produced. For example, people with aphasia after stroke may produce words that are short in length during *animal fluency*, due to difficulties at the lexical level (Faroqi-Shah & Milman, 2018).

A third method is *network analysis*. This method is used to estimate (from fluency data) which word pairs are similar based on the probability of co-occurrence of those words in a fluency task. To clarify, given the frequency of appearance of the words in a fluency task, nodes of the network correspond to the words produced, and edges between node pairs correspond to whether the words appear near each other (Goñi et al., 2011; Nevado et al., 2021; Zemla & Austerweil, 2018; Zemla et al., 2020). These edges can be compared in category and subcategory networks to see whether associations in category fluency are the same as those in subcategory fluency. It is also possible to examine the structure of the network itself using metrics, such as average shortest-path length (ASPL), cluster coefficient, or average node degree, that quantify the structure of the category’s representation (Castro & Vitevitch, 2023; Zemla & Austerweil, 2019).

Finally, a new method is to *generate words with large language models (LLMs)*. Recent work has compared fluency data generated by humans with fluency data generated by LLMs (Qiu et al., 2025; Wang et al., 2025) or transformer-based language models (Nighojkar et al., 2022). Specific to fluency tasks, Wang et al. (2025) used transformer-based LLMs (GPT-3.5-Turbo, GPT-4, LLaMA-2-70B, LLaMA-3-8B, LLaMA-3-70B) to generate English animal fluency data. The LLM-generated data were compared with the results of 30 participants from a published study (Zemla et al., 2020). Their data indicated that humans produced more unique words, although the total number of words produced was significantly lower or higher depending on the language model. Wang et al. (2025) also derived network metrics and found larger ASPL, larger modularity, and smaller cluster coefficient in LLM-generated networks than that of humans. The authors interpreted these results from the perspective that LLMs reproduce “viewpoints or expressions” rather than truly reflect the mind’s organization of the semantic system. Also, authors suggested that current LLMs have “poor associative ability and creativity.” Therefore, LLM-generated data should be used with caution. Two other points worth discussing about this study are that the authors compared human data from and LLM-generated data of 30 “participants” with different occupations (e.g., police officer, teacher, doctor, college student). Also, some of the data was generated with a maximum level of randomness and creativity (i.e., temperature)—a parameter which could be too distant from what humans would do, even though there exist no current benchmarks for this factor (Jain et al., 2023; Peeperkorn et al., 2024). In another study, Qiu et al. (2025) showed that LLM-generated data for letter fluency tended to have words that were less diverse, shorter, and more frequent than that generated by humans. The same authors also looked at networks metrics and showed that human networks were more tightly integrated (i.e., higher clustering coefficient and longer average shortest-path length). In some of these studies, the generation of words with LLMs can be seen as part of “machine psychology”—a type of research that uses behavioral experiments typically applied in humans to understand how computers work (Abramski et al., 2025; Binz et al., 2025; Liesenfeld et al., 2023; Warstadt et al., 2023). In this paper, our goal is however slightly different: It is not our intention to study whether LLMs and humans respond to fluency tasks in the “same way.” Rather, we use LLM data to understand whether LLMs can help us generate hypotheses to be tested on human participants.

### Aims and predictions

Our aim is to understand whether searches in the lexico-semantic system during category fluency tasks differ from subcategory fluency (e.g., differences in productivity, how items group together, the types of items produced); whether these differences are robust across contexts (e.g., *animals* vs *farm animals*, *foods* vs *fruits*, *transport* vs *bike parts*); and whether the associations that guide lexical search in category and subcategory fluency task are the same (e.g., similar clusters are produced in category and subcategory fluency, word network overlap).

In Study 1, we first explore whether there are differences between category and subcategory measures of fluency productivity (number of correct words) and how items group together (number of cluster switches and average cluster size). We predicted people will produce a higher number of words for category fluency compared with subcategory fluency, because category fluency represents a less constrained pool of words than subcategory fluency. We also predicted fewer cluster switches for subcategory than category fluency, which follows if people generate fewer words overall. However, we did not make predictions about average cluster size, in part because very few studies have examined subcategory fluency and it is not certain which attributes people will use to cluster responses in these tasks. We also expected these measures to vary across categories (*animals*, *foods*, *transport*) because they vary in size and structure.

We then explore whether there are differences in the properties of words generated in category and subcategory fluency (word frequency). We predicted there would be lower frequency words for subcategory fluency than in category fluency. This is expected because the more restricted set of words in subcategory fluency are likely to have fewer generics and may be more specialized. We also examined orthographic/phonological word properties (word length, orthographic neighborhood density, and phonological neighborhood density) but did not predict any differences between category and subcategory.

Finally, we explore whether people group words in subcategory fluency differently than in category fluency. We do this in two ways: one way is to examine the subcategory members generated within category fluency, and see whether the average cluster size for these items (using the subcategory clustering scheme) is similar to the average cluster size in subcategory fluency. In other words, if people cluster farm animals by function (e.g., used for fur, used for meat) in subcategory fluency, do they also cluster farm animals this way in category fluency? The second way is to construct networks for each subcategory that is a strict subset of its category, and compare the extent to which these networks overlap. For example, we compared two networks of *farm animals*, one generated from *farm animal fluency* and the other generated from *animal fluency*. If associations are consistent, we expect most edges in the category network to also exist in the subcategory network, so long as the nodes exist in both networks. If associations are inconsistent (i.e., if context matters), we expect there to be some shared edges, but many others that are unique to only the category or subcategory network.

We acknowledge that the subcategory *bike parts* has a different relation to its category *transport* compared with the other categories (*animals* and *farm animals*, *foods* and *fruits*). Therefore, the pattern of results for the latter pairs may not generalize to *transport* versus *bike parts*. If this is the case, the results could tell us whether differences in category and subcategory fluency hinge on the subcategory being a strict subset, or whether the results generalize to semantically related categories with fewer members but are not strict subsets.

In Study 2, we generated LLM data to simulate the results of Study 1. The predictions of Study 2 are the same than in Study 1 regarding differences between category and subcategory fluency. Analyses of network metrics are not performed in Study 2.

## Study 1: Category and subcategory fluency in healthy young adults

### Participants

Forty-eight Dutch-speaking young adults took part in this study. Participants consisted of 36 women and 12 men. One participant was excluded because the data were lost due to a technical error (i.e., the audio file was deleted before transcription) who received at least 15 years of education (*M* = 18, *SD* = 1.64). The age range of the participants varied from 20 to 30 years old (*M* = 23, *SD* = 2.68). All subjects participated voluntarily and consented for anonymous processing of their results for scientific purposes. Ethics approval to perform this study was obtained from the Ethics board of the Faculty of Arts at the University of Groningen (CETO).

### Materials and procedure

Participants responded to six fluency tasks (1 min per task). For category fluency, the categories were *animals, transport* and *foods*. Each category had a corresponding subcategory, which we deemed to be semantically related based on world knowledge. The corresponding subcategory for *animals* was *farm animals*, for *transport* it was *bike parts*, and for *foods* it was *fruits*.

Each session took approximately 20 min. The first part included obtaining informed consent and information about the participant’s age, sex, and years of education. Then, a short instruction about the tasks followed. Participants were asked to name as many words as possible for a certain category. To verify whether the participant had understood the instruction, the category *clothing* was used as a practice trial. After the participant named approximately five to ten words, the actual experiment could be continued. It was allowed to repeat the instruction once again when asked for by the participant. After the instructional part, participants were given 1 min to name as many words as they could think of for a certain category. Each participant was presented with the categories in a different order, following a Latin-square design. Subcategories were never presented adjacent to their corresponding category to avoid priming effects. When the participant began to speak, the experimenter started the audio recording, and a stopwatch was used to keep time. The exact instructions can be found in Appendix A.

### Scoring

#### Correct words

Participant recordings were used to transcribe their fluency responses verbatim in a score sheet. After that, words were marked as correct or incorrect by one of the authors (D.v.D.) based on a series of criteria. Doubts on the use of these criteria or questions were discussed with a senior author (A.R.). Specifically, repetitions of words that had already been mentioned were scored as incorrect. Synonyms and brand names were also scored as incorrect. Next to these general scoring principles, specific scoring principles were determined for some of the categories and subcategories. For *animals*, sex-specific and age-specific names of the animal species were all counted as correct (e.g., cow, bull, calf). The same applied to *farm animals*. Animals living in a petting zoo (e.g., goat, wild boar) and animals that are associated with a farm (e.g., cat, dog, mouse) were also scored as correct in this subcategory. For *foods*, herbs and aromatics were excluded but dishes on the other hand dishes were included (e.g., Bami Goreng, Spaghetti Bolognese). Finally, only parts that are inherent to a bicycle were considered as correct in the subcategory *bike parts.* In other words, accessories such as “panniers,” “stickers,” or a “child seat” were marked as incorrect.

#### Number of cluster switches and average cluster size

Number of cluster switches and average cluster size were calculated on the correct words with SNAFU (Zemla et al., 2020). SNAFU requires specific schemes for each category fluency task. In the schemes, the experimenter can indicate how words belong to specific categories (e.g., “cow” as a farm animal, “apple” as fruit). Cluster size is indicated by the number of successive responses that share a single subcategory (e.g., “fruits” within *foods fluency*). For example, in the series “hamster, cat, dog, wolf, coyote, zebra”, the words “hamster, cat, dog” are a cluster of size three because they are all pets, but “wolf” is not. The average cluster size is a value representing how big are all clusters produced by the participant, given a specific task. A switch represents an instance where the participant produces a response that is not part of the previous cluster.

In this paper, we used six schemes to measure number of cluster switches and the average cluster size. The schemes and the specific categories of each scheme can be found in the Appendix B. For animal fluency, we used a Dutch adaptation of the animal scheme provided in original SNAFU publication (Zemla et al., 2020). The scheme was used in a recent publication from our group (Rofes et al., 2023) and is available for download (github.com/jmjvonk/2022_Rofes_SMART-MR ). This animal scheme is based on original categorization of Troyer et al. (1997), which was later augmented by Hills et al. (2012) and then by Zemla et al. (2020). It contains categories such as African animals, birds, and feline. For the other fluency tasks, we created five new schemes which are available for download (https://osf.io/gr6cd/).

The creation of these five new schemes started by scrutinizing the words that participants used in each of the fluency tasks we report in this manuscript. A.R. and D.v.D. came up with labels for each fluency task and attributed a preliminary label to each of the words. For *farm animals* (*N* = 102 words), we used labels to indicate how we benefit from the animals, be it by the products we obtain from them or the use we make to support humans (i.e., dairy & meat, fur & fiber, pets, poultry & birds, and work). For *foods* (*N* = 135 words), we divided the words based on food groups (i.e., dairy, fats and oils, fruit, grains, vegetables, and protein). For *fruits* (*N* = 69 words), we divided the items based on nutritional content (i.e., berries, citrus, melon, pome, stone, and tropical). For *transport* (*N* = 121 words), we divided the items based on element and purpose (i.e., land, space, water, and specialized). For *bike parts* (*N* = 59), we had the following categories: brakes, drivetrain, frame, handlebar, and wheels and tires.

We then ran five online closed card sorting experiments. In a closed card experiment, participants read a written word (e.g., “donkey”) and indicate to which of a series of predetermined categories (i.e., hence “closed card”) the written word belongs to (“dairy and meat,” “fur and fiber,” “pets,” “poultry and birds,” “work,” “other”). For this specific example, participants may select “work,” since donkeys have been traditionally used as to transport goods and people, as well as in farming and construction (e.g., Davis, 2019). We recruited 22 healthy native Dutch speakers via the online research platform Prolific (seven women; mean age = 28 years, *SD* = 4; mean education in years = 17, *SD* = 1). In each experiment, participants read and categorized words (one word at a time). Before the experiment started, we provided an explanation of each label along with one example (e.g., “dairy”: foods made from milk, such as cheese). In all experiments, participants were given the option to select the label “other.” All participants responded to all five closed card sorting experiments. The experiment was hosted in the University of Groningen JATOS server (free, open-source platform). The experiment materials and data can be found in the repository (https://osf.io/gr6cd/).

The online closed card sorting experiments allowed us to attribute objective labels to the words in each of the cluster schemes. To do so, we first checked the label that participants chose most often for each word (e.g., for “donkey” the label most used was “work” and 73% of participants did so). After that, we contrasted the label that participants most often selected with our predetermined choice (i.e., for “donkey” we expected “work”). If the label most chosen by participants in the card sorting test differed from our preliminary judgment (this occurred for 1% of items), we changed the label to that indicated in the closed card sorting experiment (e.g., for “fish” we chose “dairy and meat,” and indeed some 31% of participants chose it. However, the label that was most often selected was “pet” 36% of the time). As exceptions, we selected the second most chosen label when participants most frequently chose “other” in the card sorting experiment. Participants were informed to choose “other” are when they were unsure of which label was most appropriate (e.g., many participants chose “other” for “kiwicha,” a type of grain). For the category “foods,” we kept our predetermined judgment for “vegetables,” since due to a coding error this label was not included in the card sorting test.

To judge whether the labels we chose are adequate to classify the words, we checked the percentage of words that belonged to each category after objectizing the labels (i.e., checking that the preliminary label established by A.R. and D.v.D. matched or not the results of the card sorting test). Also, we checked how often people agreed, given the words of the card sorting test, that the category was adequate to define a word (e.g., for fur and fiber, we obtained a score of 48.5 by averaging that 82% people agree with the item “sheep” belonging to this label, 54% agree with this label for “alpaca,” 45% for “llama”). The average score across the different labels regarding how often people agreed that individual words belonged to each category was 73 (*SD* = 16), which we regard as adequate. Some of the labels that people agreed the most on were “brakes” for the task bike parts (98.9 score and 13.6% of words in the *bike parts* card sorting task), “water” for the task transport (98.4 score and 19.6% of words in the *transport* card sorting task). Contrarily, labels that people agreed the least on were “frame form” of the task bike parts (40.9 score and 28.8% of words in the bike part sorting task) and “fur and fiber” of the task farm animals (48.5 score and 8.9% of words in the farm-animal task). A table with the results of these analyses can be found in Appendix C. This labelling has limitations which we go back to in the discussion.

##### **Word properties**

Five word properties were extracted for each word counting toward the total score: SUBTLEX-NL (Keuleers et al., 2010) was used to extract logarithmic frequency values for each word (i.e., log10(FREQcount + 1), where FREQcount represents the number of times the word appears in the corpus). This database is a corpus of subtitles from Dutch-speaking TV-shows and movies and includes more than 43 million words. CLEARPOND (Marian et al., 2012) was used to obtain phonologic and orthographic neighborhood density. In this database, neighborhood density is calculated by counting the number of words (based on SUBTLEX-NL) differing from the target word by adding, deleting, or substituting one grapheme/phoneme. Finally, we measured word length by counting the number of graphemes of each correct word.

To minimize the number of empty values in the database, if a generated word could not be found in the databases, we searched for the value of an alternative form of the word in the same database. For words that could not be found in the database were replaced by synonyms (e.g., *mot* “moth”–*nachtvlinder* “night-moth”). When this method did not return a value, we imputed the value (mean score per participant for each word property). The number and percentage of imputed values for each property were: frequency (387/5305, 7.3% missing values), orthographic neighborhood (1295/5305; 24.4% missing values), phonological neighborhood (1295/5305; 24.4% missing values), word length in graphemes (0% missing values).

##### **Word networks**

For each category and subcategory, word networks were estimated using the conceptual network method (Goñi et al., 2011) implemented in SNAFU (Zemla et al., 2020). This method treats all responses as nodes in the network, and estimates an edge between any two nodes when the two responses co-occur (within a window of plus or minus two words) significantly more than expected by chance across all lists for that category or subcategory. For descriptive purposes, we calculated the following metrics on each network: clustering coefficient, density, number of edges, number of nodes, average node degree, average shortest path length, and diameter. We then used Cytoscape to plot the networks, plotting only nodes that had at least one edge.

### Analyses

We performed analyses at two different levels. First, we compared category versus subcategory fluency lists directly (“Differences between category and subcategory fluency”). One limitation of these analyses is that the pool of valid words is larger for category than subcategory fluency. To address this, we then conducted additional analyses that are restricted only to valid subcategory responses (“Subcategory responses in each corresponding category fluency”). For example, we assessed whether valid subcategory responses were clustered similarly in both category and subcategory fluency.

#### **Differences between category and subcategory fluency**

To assess whether there are differences between category and subcategory fluency tasks we ran multiple regression models with the following dependent variables: correct words, word frequency, orthographic neighborhood, phonological neighborhood, word length in graphemes, number of cluster switches, and average cluster size. We included an interaction factor for two independent variables: *group* (a binary factor indicating whether the tasks corresponded to category or subcategory fluency) and *field* (a tripartite factor indicating whether the tasks required producing animals, foods, or transport—regardless of whether they were produced in a category or subcategory fluency tasks). We also included a random intercept per participant, to take into account inter-individual differences (e.g., glmer(Words_correct ~ group * field + (1 | ID), family = “poisson”, data = data)). Random slope estimates for group and field were not included due to convergence issues. To assess whether the differences emerged when comparing pairs of fluency tasks that are related (e.g., animals vs farm animals), we ran regression models with the same dependent variables, the independent variable *group*, a random intercept per participant (e.g., glmer(Words_correct ~ group + (1 | ID), family = “poisson”, data = data[field == “animals”]). Random slope estimates for group and field were not included due to convergence issues. We analyzed each of *fields* one at a time. For these analyses each SNAFU scheme is paired with its category or subcategory (e.g., we applied the farm animal scheme to farm animal fluency). We assessed whether the model residuals met the assumption of normality using the Shapiro–Wilk test and Q–Q plot visualizations. When possible, we applied data transformations (logarithmic or square root) and proceeded with linear mixed-effects models (*lmer*) if normality was achieved. For count variables, or if normality could not be achieved through transformation, we fitted generalized linear mixed-effects models (GLMM; *glmer*) with an appropriate distribution (typically Poisson for counts, Gamma for continuous) instead of a linear mixed-effects model. *P* values were calculated using the *lmerTest* package, which uses the Satterthwaite method to approximate the degrees of freedom for each statistic. *F* statistics for omnibus tests were obtained by passing the *lmer* model to the “anova” function in R. For the GLMMS, we only reported *p* values for the fixed effects using likelihood ratio tests (Drop1).

These analyses were complemented with analyses of network metrics. For each network, we reported common network metrics (i.e., cluster coefficient, density, number of edges, number of nodes, average node degree, average short path length). We then compared the values between subcategory and category networks (excluding *transport* and *bike parts*).

#### Subcategory responses in each corresponding category fluency

Second, we studied words belonging to subcategory fluency within each corresponding category fluency task. That is, we aimed to understand whether people group words in subcategory fluency differently than in category fluency. To do so, we used the subcategory scheme to calculate the average cluster size within the superordinate category fluency. We then compared these to the average cluster sizes in subcategory fluency. For example, if people cluster “dairy and meat” animals together in *farm animal* fluency, do they also cluster them together in *animal fluency*, or do they simply cluster “farm animals” irrespective of their function on a farm? For these analyses, we excluded *transport* and *bike parts* fluency, since none of the valid responses in *transport* fluency can be coded as *bike parts* (i.e., handlebar or saddle are not transportation means). We also compared the number of valid subcategory responses in both category and subcategory fluency (e.g., the number of farm animals listed in *animal fluency* vs farm *animal fluency*).

These second type of analyses were complemented by analyses of network metrics. We reported the number of shared and unique edges among shared nodes between networks. For example, *koe* (cow) and *kip* (chicken) appear in the subcategory network farm animals and in the category network animals, and in both networks the same nodes share an edge; *eend* (duck) and *gans* (goose) appeared in both networks as well but were joined by an edge in only the subcategory network for farm animal. In addition, we reported the number and percentage of edges that were categorized as similar in each scheme. For example, *kip* (chicken) and *haan* (rooster) were categorized as “poultry birds” in the farm animal scheme, but *kip* (chicken) and *haas* (hare) were not categorized together in the same scheme (*haas* is categorized under “pets”). Finally, we conducted a bootstrap analysis to determine whether the number of shared edges is less than expected by chance, which would indicate reliance on distinct associations in category and subcategory fluency tasks.

### Results

The raw data and some of the scripts can be accessed online (https://osf.io/gr6cd/).

#### Differences between category and subcategory fluency

Table 1 includes descriptive statistics for the variables *group* (categories, subcategories) and for each task.

**Table 1:** Descriptive statistics per task group (category, subcategory), and per individual task for correct words, word properties, average cluster size and number of cluster switches. Human data (Study 1)

Task	Corr.Words	Frequency	Phon.Neigh	Orth.Neigh	Length	N.switches	Cluster size
Category	21 (7)	2.39 (.32)	6.64 (2.45)	4.78 (1.84)	6.62 (.85)	5.41 (3.64)	2.64 (1.21)
Subcategory	15 (4)	2.18 (.43)	8.12 (4.65)	6.04 (3.41)	6.88 (1.28)	7.78 (2.21)	1.59 (.34)
Animals	25 (6)	2.4 (.19)	8.43 (2.28)	6.56 (1.51)	6.06 (0.73)	9.56 (2.72)	2.49 (.63)
Farm animals	13 (3)	2.71 (.21)	13.6 (2.2)	10.16 (1.6)	5.47 (.89)	7.9 (1.67)	1.49 (.27)
Foods	23 (7)	2.09 (.21)	5.68 (1.85)	3.66 (1.23)	6.96 (.71)	3.27 (1.89)	2.22 (1.18)
Fruit	15 (4)	1.77 (.1)	3.17 (.87)	2.61 (.81)	7.58 (.68)	8.73 (2.35)	1.62 (.35)
Transport	15 (4)	2.69 (.22)	5.83 (2.17)	4.1 (1.21)	6.84 (.82)	3.39 (1.73)	3.21 (1.46)
Bike parts	17 (4)	2.07 (.22)	7.6 (2.05)	5.33 (1.53)	7.59 (.84)	6.73 (2.13)	1.66 (.36)

Corr.Words = number of correct words; Phon.Neigh = phonological neighborhood, Orth.Neigh = orthographic neighborhood;

Length = length in graphemes; Cluster Size = average cluster size (static); N.Switches = number of cluster switches (static).

##### **Correct words**

**Fig. 1:**
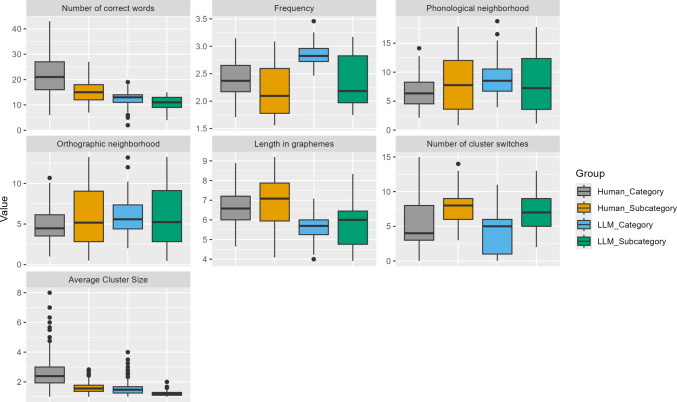
Correct words and word properties (category vs subcategory, human and LLM-generated data). (Color figure online)

We found an interaction between *group* and *field,* χ^2^(2) = 124.67, *p* <.001. Further analyses revealed that participants produced more correct words in the category task *animal fluency* (*M* = 25, *SD* = 6) than in the subcategory task *farm animals* (*M* = 13, *SD* = 3), χ^2^(1) = 181.48, *p* <.001, and they produced more correct words in the category task *foods* (*M* = 23, *SD* = 7) than in subcategory task *fruit* (*M* = 15, *SD* = 4), χ^2^(1) = 70.969,* p* <.001. However, they produced more words for in the subcategory task *bike parts* (*M* = 17, *SD* = 4) than in the category task *transport* (*M* = 15, *SD* = 4), χ^2^(1) = 6.299, *p* =.001 (see Fig. 1).

##### **Number of cluster switches and average cluster size**

We found interactions between *group* and *field* for both number of cluster switches, *F*(2, 235) = 80.745, *p* <.001, and average cluster size, χ^2^(2) = 168.83, *p* <.001.

**Fig. 2:**
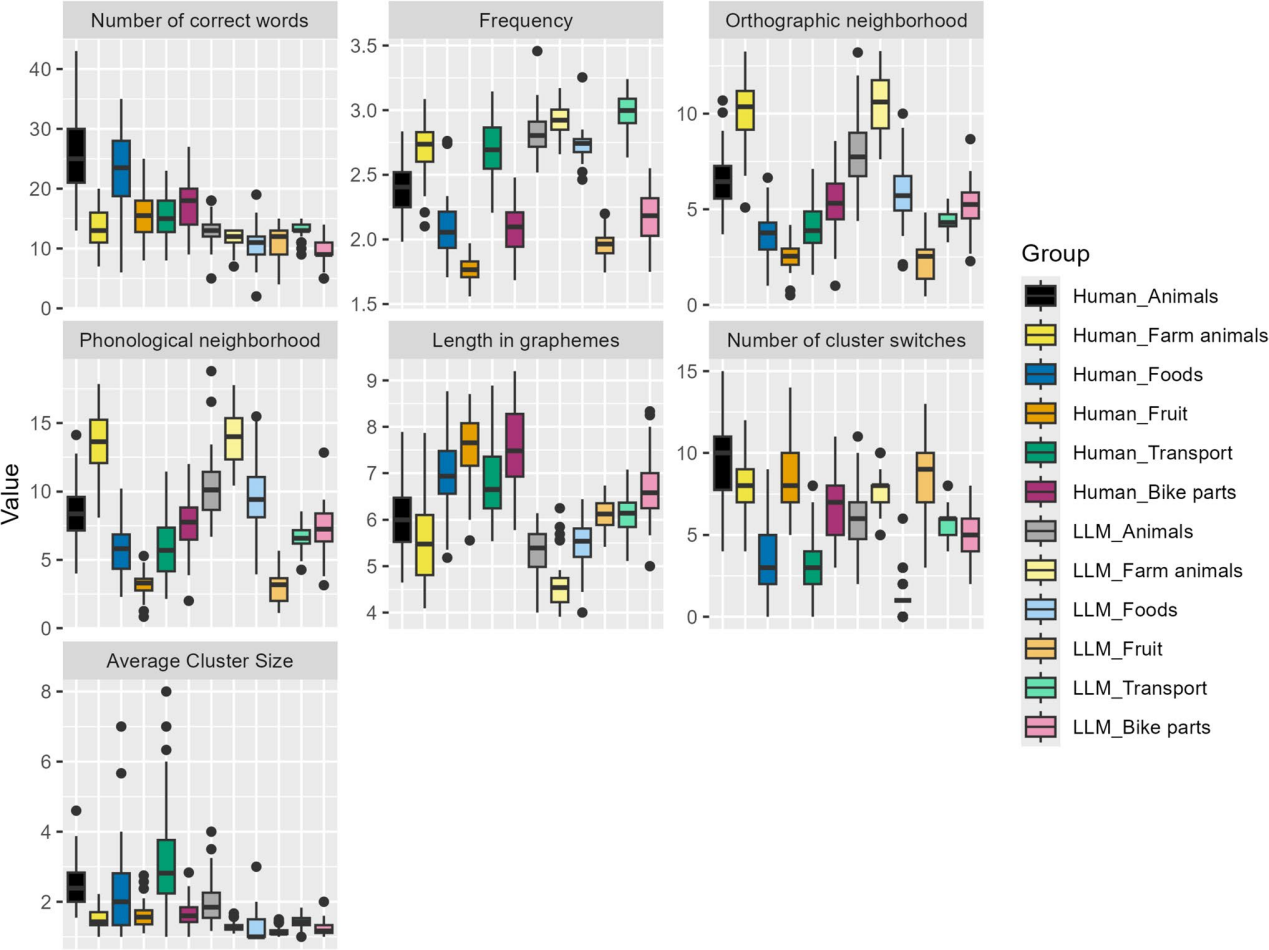
Correct words and word properties (per task, human and LLM-generated data). (Color figure online)

Further analyses revealed that participants produced fewer switches in the category fluency tasks *foods* (*M* = 3.27, *SD* = 1.89) and *transport* (*M* = 3.39, *SD* = 1.73) compared with the subcategory fluency tasks *fruits* (*M* = 8.73, *SD* = 2.35), *F*(1, 47) = 168.91, *p* <.001, and *bike parts*, respectively (*M* = 6.73, *SD* = 2.13), *F*(1, 47) = 101.62, *p* <.001. However, participants produced more switches in the category fluency task *animals* (*M* = 9.56, *SD* = 2.72) than the subcategory fluency task *farm animals* (*M* = 7.9, *SD* = 1.67), *F*(1, 47) = 17.669, *p* <.001 (see Fig. 2).

Regarding cluster size, people produced bigger clusters for the category fluency tasks *animals* (*M* = 2.49, *SD* =.63), *foods* (*M* = 2.49, *SD* = 1.55), and *transport* (*M* = 4.53, *SD* = 2.54), than for the subcategory fluency tasks *farm animals* (*M* = 1.7, *SD* =.31), χ^2^(1) = 110.17, *p* <.001, *fruits* (*M* = 1.67, *SD* =.38), χ^2^(1) = 19.144, *p* <.001, and *bike parts* (*M* = 1.69, *SD* =.42), χ^2^(1) = 87.81, *p* <.001.

##### **Word properties**

We found interactions between *group* and *field* for all word properties (see Table A1, Appendix). Relative to word frequency, participants produced more frequent words for the category fluency tasks *foods* (*M* = 2.09, *SD* =.21) and *transport* (*M* = 2.69, *SD* =.22) than for the respective subcategory fluency tasks *fruits* (*M* = 1.77, *SD* =.1), χ^2^(1) = 83.432, *p* <.001, and *bike parts* (*M* = 2.07, *SD* =.22), χ^2^(1) = 132.38, *p* <.001. However, people produced words in the subcategory task *farm animals* (*M* = 2.71, *SD* =.21) that were more frequent than in the category task *animals* (*M* = 2.4, *SD*=.19), χ^2^(1) = 71.498, *p* <.001.

Regarding orthographic neighborhood and phonological neighborhood, we found the same pattern: participants produced words with fewer orthographic/phonological neighbors for the category fluency tasks *animals* and *transport* than for to the subcategory fluency tasks *farm animals* and *bike parts*, respectively (Table 1, Table A1). However, participants produced words in the category fluency *foods* that had more orthographic/phonological neighbors than the subcategory *fruits* (Table 1, Table A1).

Regarding word length, participants produced longer words for the subcategory tasks *fruits* (*M* = 7.58, *SD* =.68) and *bike parts* (*M* = 7.59, *SD* =.84) compared with the category tasks *foods* (*M* = 6.96, *SD* =.71), *F*(1, 47) = 33.445, *p* <.001, and *transport* (*M* = 6.84, *SD* =.82), *F*(1, 47) = 29.006, *p* <.001. However, people produced longer words in the category fluency task *animals* (*M* = 6.06, *SD* =.73) than in the subcategory fluency task *farm animals* (*M* = 5.47, *SD* =.89), *F*(1, 47) = 19.463, *p* <.001 (see Fig. 2).

##### **Word network metrics**

**Fig. 3:**
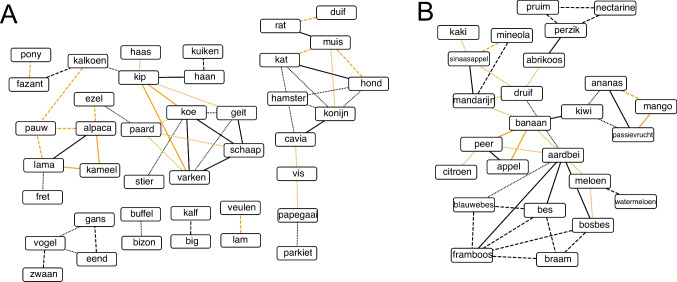
Word networks (category, subcategory, and per task). Plotted are nodes shared in both networks (category and subcategory) that contain an edge in at least one network for (**A**) animals and farm animals and (**B**) foods and fruits. Solid line = edge in both networks; dotted line = category network edge only; dashed line = subcategory network edge only. Black edge = two nodes appear in the same cluster in SNAFU scheme; orange edge = two nodes do not appear in the same cluster in SNAFU scheme. (Color figure online)

Table 2 includes descriptive statistics for network metrics and Fig. 3 includes plots of each of the networks. From a descriptive standpoint, *category fluency* has a lower cluster coefficient, lower density, lower average node degree, and greater number of edges, number of nodes, average shortest-path length (ASPL) and diameter. This same pattern can be found for the category fluency *animals* versus the subcategory fluency *farm animals*, and for the category fluency *foods* versus the subcategory fluency *fruits*. The comparison between category fluency *transport* and subcategory fluency *bike parts* is the same for all factors, except the cluster coefficient. Also, the differences in density, number of edges, number of nodes, average number degree, ASPL, and diameter are less marked than for the other tasks.

**Table 2:** Word network metrics. Human data (Study 1)

Task	Clustering coefficient	Density	Number edges	Number nodes	Average node degree	ASPL	Diameter
Category	0.131	0.003	541	605	2.865	6.186	14
Subcategory	0.212	0.015	338	216	4.549	5.482	12
Animals	0.125	0.006	132	213	1.732	5.170	11
Farm animals	0.141	0.016	32	64	1.132	2.357	5
Foods	0.097	0.003	132	278	1.406	7.425	16
Fruits	0.204	0.017	33	62	1.227	2.429	5
Transport	0.040	0.005	41	125	0.814	2.867	6
Bike parts	0.044	0.007	30	90	0.748	2.786	6

ASPL = average short path length.

#### Subcategory responses in each corresponding category fluency

##### **Average subcategory cluster size in category and subcategory fluency**

Participants listed more farm animals in *farm animal fluency* (*M* = 13.41, *SD* = 3.04) than *animal fluency* (*M* = 10.93, *SD* = 3.78), *t*(92.95) = 3.78, *p* =.001. They also listed more fruits in *fruits fluency* (*M* = 15.39, *SD* = 39) than *foods fluency* (*M* = 4.48, *SD* = 3.88), *t*(93.95) = 13.81, *p* <.001. Average farm animal cluster size was not different in both *farm animal fluency* (*M* = 1.49, *SD* =.27) and *animal fluency* (*M* = 1.49, *SD* =.31), *t*(91.87) =.085, *p* =.93. However, participants produced larger fruit cluster sizes in *fruit fluency* (*M* = 1.61, *SD* =.35) than *foods fluency* (*M* = 1.26, *SD* =.26), *t*(83.51) = 5.321, *p* <.001.

##### **Overlap between category and subcategory networks**

The *animal* and *farm animal* networks shared 45 nodes, accounting for 70% of the nodes in the *farm animal* network (45 of 64). Among these shared nodes, the vast majority of pairs were not associated in either network (940 nonedges). However, 50 pairs formed an edge in at least one network: 13 pairs were linked in both networks, 14 in the *farm animal* network only (36% belonged to the same cluster), and 23 in the *animal* network only (67% belonged to the same *farm animal* cluster). The *fruits* and *foods* networks shared 35 nodes, accounting for 56% of the nodes in the *fruits* network (35 of 62). Again, the vast majority of pairs among these shared nodes did not form an edge in either network (555 nonedges). However, 40 pairs formed an edge in at least one network: 11 shared in both, 14 unique to the *fruits* network (86% belonged to the same cluster), and 15 unique to the *foods* network (29% belonged to the same fruits cluster).

The edges in these networks reflect the mental associations that guide participant responses in the fluency tasks. Both category and subcategory networks are sparse (few edges). The networks agree on the absence of edges for most node pairs, but at the same time less than 50% of the edges in one network were present in the other (after controlling for shared nodes). We conducted a bootstrap analysis to determine the level of overlap that is expected from chance alone. Our approach is identical to one used in prior work to assess differences in network structure across two populations (Nevado et al., 2021; Wulff et al., 2022). First, we randomly permuted the labels (category or subcategory) on each fluency list. Then, we estimated “subcategory” networks and “category” network from these bootstrap samples, though in reality each network was formed from a mixture of data from both tasks. Since the data are pooled, any difference in network structure is due to chance alone. As above, we extracted only the shared nodes between them for comparison, and then calculated the proportion of shared edges relative to the total number of edges (i.e., the number of shared edges plus the number of edges unique to each network). This process was repeated 1,000 times to form a distribution, indicating the proportion of shared edges that is expected from chance alone (i.e., if there were no difference in the associations used for category and subcategory fluency).

The proportion of shared edges for the *animal* and *farm animal* networks was .26 (13 shared edges over 50 unique edges, as described above). This fell within the 95% confidence interval of what is expected by chance, CI [.240,.333], *p* =.263. The proportion of shared edges for the *fruits* and *foods* networks was .275 (11 shared edges over 40 unique edges). This fell within the 95% confidence interval of what is expected by chance, CI [.217,.301], *p* =.41.

## Study 2: Category and subcategory fluency generated by a large language model (LLM)

In a second study, we used a local instance of the Mistral-Small-24B-Instruct-2501-Dutch model (Mistral AI Team, 2025) to generate fluency data. The LLM was given the same instructions as human participants, and we generated data for 50 “pseudo-participants.” The data were analyzed identically to the human data, except that no network analyses were conducted. We conducted the same set of statistical analyses as in Experiment 1 in order to see whether variables that influence human fluency also affect LLM fluency. These analyses provide a sense of whether LLM fluency data can be used to predict which effects might be significant with human participants. Extensive information regarding the methods, analyses, and results of this study can also be found in Appendix E. We focus our discussion here on the similarities and differences between human and LLM fluency data (i.e., whether the same analyses that were significant in Study 1 were also significant for Study 2), but note that the two data sets were not compared in the same statistical model.

We did not directly compare LLM data to human data, as this was outside the scope of the paper. That said, descriptive statistics and correlations between human and LLM data are provided in the Appendix for interested readers (Tables A2–A5, Fig. A1). To help us interpret the results obtained in Study 2, we performed a post-hoc comparison of lexical diversity between the LLM data and the human data, similar to other studies (Cunningham & Haley, 2020; Qiu et al., 2025; Rofes et al., 2018; Schepens et al., 2025; Wang et al., 2025). We calculated the total number of tokens (i.e., total number of words produced), total types (i.e., number of distinct words), percentage of hapax types (i.e., percentage of types that appear only once in each dataset), type-token ratio (TTR; number of types divided by number of tokens), mean TTR with a sliding window of 100 words (commonly used), and mean TTR with a sliding window of 15 words (approximate average of words produced in verbal fluency tasks per participant). Mean TTR measures are useful because the datasets in our study are expected to have different number of tokens and types.

### Similarities between human and LLM generated fluency data

Regarding the differences between category and subcategory fluency, we found similar results between the human sample and the LLM-generated data for all word properties (word frequency,

orthographic and phonological neighborhood, word length) and average cluster size (Fig. 1). That is, all statistical tests that were statistically significant (*p* <.05) in Study 1 were also significant (in the same direction) in Study 2, and vice versa. These results hold not only for category versus subcategory comparisons, but also within each semantic field (Fig. 2).

### Differences between human and LLM generated fluency data

That said, we also found discrepancies between the two datasets: for correct words humans produced more words for the subcategory *bike parts* than the category *transport*, while in the LLM-generated sample we found the opposite pattern (*p* <.001). Humans also produced more words for the category *foods* than the subcategory *fruit*, while this difference was not significant in the LLM-generated sample (*p* =.92). In both studies, more words were generated for *animals* than *farm animals*.

In both studies, we found fewer switches in category than subcategory fluency, however our results differed for specific fields. In humans we detected fewer switches in *bike parts* than *transport,* and more switches in *animals* than *farm animals*. In the LLM data we found the opposite patterns (*p* =.019 for *transport* and *p* <.001 for *animals*). The results were consistent between studies for *foods* and *fruits*.

Regarding average subcategory cluster sizes in each corresponding category fluency, the results obtained in humans and the LLM-generated were the same, with the exception of average fruit cluster size, where humans produced larger fruit cluster sizes for the subcategory *fruit* compared with the category *foods*, while the LLM-generated data indicated a lack of overlap for fruit clusters between *foods* and *fruits* (only two fruits were generated by the LLM when prompted to list foods)*.*

### Lexical diversity between human and LLM generated fluency data (all tasks)

The human dataset contained 5,355 tokens and 1,100 types, with a TTR of 0.205. The LLM simulation had 3,813 tokens and 570 types, with a lower TTR of 0.149, but a higher percentage of hapax types (54.39% vs 49.55%). For mean TTR, the human dataset had a value of 0.924 with a 100-word sliding window. The LLM generated data showed a lower Mean TTR of 0.831 for the 100-word sliding window. Both datasets showed high mean TTR values (close to 1) for the 15-word window. Table A5 (Appendix) includes all lexical diversity measures.

## Discussion

In this paper, we set out to investigate whether searches in the lexico-semantic system during category fluency tasks differ when context is constrained (superordinate category vs subcategory); whether these differences are consistent across different semantic fields (*animals* vs *farm animals*, *foods* vs *fruits*, *transportation* vs *bike parts*), and whether the dominant associations to guide the lexical search in category fluency are the same as subcategory fluency. We first ran a study with healthy young adults and then a simulation of the study with data generated from an LLM (Mistral 24B).

### Differences when context is constrained and different semantic fields affect performance

The results indicate that category fluency tasks differ when context is constrained and that different semantic fields affect performance. We found that people produced more words in category versus subcategory fluency (with the exception of the subcategory bike parts for which people produced more words than for the category *transport*). Also, we found differences in the number of switches, cluster size, and all the word properties between category and subcategory fluency tasks. Regardless of the specific direction of the effects and specific difference between tasks, the general difference between category and subcategory fluency tasks underscores that context matters when it comes to producing words in fluency tasks. Even though our paper may be first at specifically stressing the differences between category and subcategory fluency, there exist numerous reports indicating that healthy participants and people with different etiologies produce different numbers of words for fluency tasks with different contexts (e.g., Moreno-Martínez et al., 2016; Ventura et al., 2005).

Our findings agree with our prediction that the number of items for category fluency is greater than that of subcategory fluency. We argue this because, at least for *animals* and *foods*, category fluency represents a less constrained task than subcategory fluency (e.g., all farm animals are animals, while the opposite is not true). Similar arguments regarding the number of potential words that people can produce in different fluency tasks have been previously raised (e.g., Moreno-Martínez et al., 2016). That said, we found the opposite pattern between fluency for *bike parts* and *transport*, whereby people produced more items in the former than in the latter. Though this result was not predicted, it may be less surprising, provided that items produced in *bike parts* fluency are related to *transport* but they do not represent a strict category within transportation means. In fact, all parts of a bicycle are not transportation means, and all transportation means are not parts of a bicycle. In addition, our study was conducted in the Netherlands, where people are well known for their use of bikes.

Nonetheless, the inclusion of these two fluency tasks (*transport* and *bike parts*) in our study is relevant because the results allow us to argue that context may constrain the number and type of words produced and also how they are produced. Our results were often consistent across all three semantic fields, and when they were not, *transport* was not always the outlier. This suggests that the differences between category and subcategory fluency may not only occur when the subcategory task triggers a restricted set of items that is semantically related to the category task (e.g., *animals* vs *farm animals*, *foods* vs *fruits*), but also when the subcategory task primes for items that are semantically related to those of the category task but that do not represent a restricted set of items of the category task (e.g., *transport* means vs *bike parts*). Comparisons between further sets of category vs subcategory tasks seem needed to further support this claim.

In addition, we found larger cluster sizes in category compared to subcategory fluency, strengthening the notion that context plays a role when it comes to producing words in fluency tasks. We did not make predictions about average cluster size, precisely because this is an aspect that has been studied very little. However, Rofes et al. (2023) found that people who produced more words also produced bigger clusters, at least in animal fluency. It seems logical for people to produce bigger clusters when faced with semantic fields that have more items or where items can more easily be grouped into semantic subcategories. These salient subcategories (e.g., aquatic animals, birds) may serve as convenient cues to facilitate retrieval of other similar animals, resulting in larger clusters, compared with semantic fields that are less easily grouped into subcategories. *Animal fluency* is indeed an archetypical example of this, since there exist different species of animals, animals have different habitats (e.g., Troyer, 2000). The corresponding subcategories we used in this experiment (i.e., *farm animals*) may be more difficult to subgroup or could be subdivided in a different way. For example, the “habitat” of farm animals may be seen as less clear than that of animals (e.g., barn, shelter, pond, pasture) or because farm animals are understood in a different way than animals (e.g., purpose they serve to humans). Similar arguments could be raised for *foods fluency* given different combinations of ingredients, cooking styles, and gastronomies; and *fruit fluency* where for example the physical characteristics of the fruit (e.g., color, taste) or how they are used (e.g., pealed, baked, dried) may play a role.

Another interpretation of these results is that the clustering scheme itself (not necessarily the context of the fluency task) is driving this effect. In fluency tasks, any labelling of items has an inevitable degree of subjectiveness and any one item can be under more than one label (e.g., a word like “fish” can be considered a “pet” but also “meat”). Also, in our SNAFU schemes, we count with over 25 groups in *animal fluency*, while only five groups for *farm animal fluency*; and in *foods fluency* we created five groups, while *fruit fluency* had six possible groups. To the best of our knowledge, there are no established thresholds to determine whether a label should be considered in a cluster scheme and how many possible groups there should be in a cluster scheme. In fact, in fluency tasks, only two words are sufficient to create a cluster. In general, we believe that labels that appear very seldomly are probably too specific to represent a cluster that most people may use to generate words during a fluency task. However, in this paper we decided to provide information regarding the labels we used to determine clusters, while waiting for replication.

We also found that in general people produced fewer switches for category fluency than subcategory fluency. This finding was unexpected and we originally predicted that the opposite would be true provided that producing more words tends to be conducive of producing more switches, at least in *animal fluency* (e.g., Rofes et al., 2021, 2023). While we did find that for *animal fluency* people produced more switches than for fluency of farm animals, these results were different for *foods fluency* vs *fruit fluency*, and for *transport fluency* vs *bike parts fluency*. Therefore, the difference between our tasks is possibly not only marked by the fact that fluency tasks require more or less general categories, but also different types of fluency tasks can play a role in these results. These differences could also be due to how we created our cluster schemes. There were fewer responses in subcategory, but also fewer cluster labels and less overlap between clusters.

We found differences between category and subcategory fluency tasks for all word properties we studied. In line with our hypothesis, people produced words in subcategory fluency (versus category fluency) that were lower in frequency. This was predicted because when the context is more specific, then words that are less common ought to be produced, while people may resolve to more frequent items (also items that may be more readily available for retrieval) when contexts are more general. One possibility is that participants subdivided category responses further in category fluency (e.g., listing only “cow” in *animal fluency*, but “Jersey cow” and “dairy cow” in *farm animal fluency*). However, when we performed a post hoc, qualitative inspection of the data we did not find much evidence for this. Only four participants produced words of “the same kind” for *farm animal fluency* such as “legkip” (laying hen) and “vleeskip” (broiler chicken), “melkkoe” (dairy cow) and “vleeskoe” (beef cow), or “herdershond” (shepherd dog) and “Shetlander” (a type of dog). This pattern also appeared in the category fluency tasks such as *transport*, particularly, with words ending in “-wagen” (wagon), such as “aanhangwagen” (trailer), “benenwagen” (on foot), “brandweerwagen” (fire truck), “feestwagen” (parade float), “kraanwagen” (crane truck), “paardenwagen” (horse trailer), and “vrachtwagen” (truck). In the *food fluency* category, we found “apple” (apple), “applemoes” (applesauce), and “appletaart” (apple pie), but these appeared only once. Overall, this pattern is not unique to subcategory fluency or a dominant strategy in fluency in our dataset.

### Dominant associations guiding lexical search

Another research question was whether people rely on the same associations to guide category and subcategory fluency production, or whether the context would affect the types of associations people rely on. For example, “horse” and “pig*”* are both farm animals and may be similar in the context of *animals* (resulting in their clustering), but when restricted to *farm animals* they have little else in common. We answered this question by examining whether subcategory responses were distributed similarly in both category and subcategory fluency, and analyzed the data using both clustering metrics and network analysis. These analyses excluded *transport* and *bike parts*, as those categories are not nested.

For the network analysis, we first constructed separate networks from category and subcategory fluency data. Descriptive measures indicated that the networks differ in structure (e.g., density, ASPL), but this may be in part because the set of responses differs between the two tasks. We then extracted a subnetwork from each that included only the nodes that were present in both networks. There was some overlap in edges between *animal* and *farm animal* networks, but each network also contained many edges unique to that network (i.e., edges between farm animals in the *animal* network that were not present in the *farm animal* network, and vice versa). It is tempting to infer that this occurs because farm animals within *animal fluency* do not mimic the same clustering structure as farm animals within *farm animal fluency*. However, a bootstrap analysis revealed that the number of shared edges did not differ from what was expected by chance, indicating that the two representations are not in fact statistically different (no evidence to reject the null). A closer inspection revealed that many edges (67%) between farm animals in the *animal* network were in fact part of the same *farm animal* cluster (e.g., “used for fur”), suggesting that the *animal* network may encode finer-level associations between animals (such as “used for fur” instead of simply “farm animals”). Similar results were obtained for *fruit* and *foods* networks.

In another approach to answer this question, we looked at the clustering of *farm animals* within *animals*, and the clustering of *fruits* within *foods.* We found that the average *farm animal* cluster size was not different in both *farm animal fluency* and *animal fluency*, but that participants generated large fruit clusters in *fruit fluency* compared to *foods fluency*. The former offers more evidence of a shared representation for the two tasks, while the latter offers some evidence of different representations that conflicts with the network analysis.

### Simulation of findings with an LLM

The simulation of findings with an LLM is somewhat encouraging as it opens the door to study other fluency tasks before they are ultimately tested in humans. While we found many similarities, we also found several differences between the two datasets and call for caution when using LLMs as a proxy for humans (e.g., in piloting studies). For example, humans produced larger fruit cluster sizes for subcategory *fruit* than category *foods.* However, this was not simulated in the LLM dataset. The difference between humans and LLMs in this regard may relate to a different “understanding” of how words/concepts relate. For example, in the *foods* category humans produced many fruits such as “strawberry,” “apple,” “pineapple,” “banana,” “berry,” “date,” “fig,” “apricot.” However, the LLM model only produced “apple” and “banana.” Regarding lexical diversity, humans produced more consistent word choices across tasks compared to the LLM model. The (mean) TTR values were closer to 1 in the human than in the LLM simulated data (TTR = 0.205 vs 0.149; mean TTR [100-word sliding window] = 0.924 vs. 0.831) indicating that humans repeated fewer words across the entire dataset compared to the LLM. These results are similar to letter fluency simulations (Qiu et al., 2025). Also, the results are relevant for the distinction between category and subcategory fluency, particularly, in the context of a study where these two types of tasks were used. For example, it could be hypothesized that participants avoid repeating words that they previously produced, sticking closely to the task instructions and/or avoiding repetition of tasks throughout the experiment (e.g., specific farm animals when producing animals, or vice versa), while the LLM model may be agnostic to task order effects. Also, the LLM-simulated data contained a higher number of unique words per “participant,” as seen by the higher percentage of types (i.e., hapax types) that appeared only once in each entire dataset (54.39% vs 49.55%). These results are different than LLM studies simulating animal and letter fluency data in English (Qiu et al., 2025; Wang et al., 2025) and other tasks in German (Schepens et al., 2025) in which the LLM models generated fewer unique words than did humans. However, our experiments were conducted in Dutch, we only used one LLM model (versus benchmarking different models), and we calculated lexical diversity on a composite measure with more than one fluency task.

### Limitations and future work

The overall findings of Studies 1 and 2 are very exciting. However, we should be careful as they have some limitations. The first limitation has to do with the comparisons between category and subcategory fluency tasks, where we collapsed the data of different tasks. These comparisons are relevant from the perspective that they provide information at a more general or abstract level than specific comparisons such as *animal fluency* versus *farm animal fluency*. However, we did not ask participants to produce words that belonged to “categories” or words that belonged to “subcategories.” In fact, such a task would have been very difficult to perform since, with the exception of free fluency tasks, in any fluency task a specific context is typically required (e.g., animals, vegetables, fruits). Consequently, the results of category vs subcategory fluency should be seen as an addition to the results between specific pairs of fluency tasks, but they should not be interpreted in isolation.

Another limitation is that category and subcategory fluency tasks can be seen as different tasks and, therefore, that comparisons between these two tasks are not meaningful. For example, we found that average cluster size is larger for *foods* than *fruits*. One possibility is that this reflects a fundamental property of search itself – people may mentally search longer within a cluster when the global set of possible responses is larger (there are more foods than fruits). But another possibility is that when you subdivide a category into clusters (fruits, meat) they will naturally contain more members than if you subdivide a subcategory into clusters (berries, citrus). In this case, context affects average cluster size, but it is a reflection of how the category itself is organized and not something related to mental search. One possibility for further work could be to reanalyze our data with cluster schemes that do not depend on human annotation (Kumar et al., 2025).

Another example could be posed for word properties. We found that people generate lower frequency words in fruit compared with foods. The same question as before could be asked: Is this reflective of mental search, or is it just that the average word frequency for all possible foods is higher than all possible fruits? While the aforementioned issue with cluster sizes and this issue with word properties are not something we can avoid with our current paradigm (or in fact with any task require spontaneous production where the items that participants need to respond to cannot be controlled for word properties, etc.), we cannot disregard that the current results may also indicate that context modulates the lexical search. If this was not the case, then it would also be hard to explain why individuals with brain damage can show dissociations between word categories, in more controlled settings (e.g., Hillis & Caramazza, 1991; Warrington & Shallice, 1984). Relative to word properties, future work could consider measures of orthographic and semantic similarity between word pairs (e.g., Levenshtein distances, cosine similarities) among the generated responses. These measures differ from the word properties we have examined (e.g., phonological neighborhood, word frequency) in that they can only be calculated between pairs of words and are not unique properties of the words themselves. However, such measures could be relevant to how words group together (e.g., similarity drop model by Hills et al. 2012; Kumar et al., 2025) which in turn could be useful to validate the new hand-coded schemes for subcategory tasks that we provided.

The question of whether context (subcategory vs category) affects mental search is difficult to answer with fluency alone. While we find differences in fluency tasks, future work may try to answer this question with other paradigms. For example, one possibility is to use stimuli similar to Tversky (1977), yet asking participants to rate the similarity between different animals versus farm animals. Participants could be asked which animal is more similar to a “horse.” In the case of the comparison between animals versus farm animals, the options could be “duck, zebra, pony” or “elephant, zebra, pony.” If people have a preference for “pony” in the first example and “zebra” in the second example, then perhaps context does play a role, and people find that items that are within one subcategory are more similar to one another than items that are in one category.

Specific to Study 2, one aspect to consider is that LLMs do not have a sense of time (e.g., Jain et al., 2023) or experience the “urge” to produce as many words as possible in 1 min. Therefore, the time pressure component that humans experience seems inexistent or it is at least not the same when generating LLM data. This time pressure is relevant in fluency tasks since, for example, switches are argued to reflect the time needed to update the criterion to generate a new subcategory (Mayr, 2002). The difference in time between humans and LLM-generated data could explain why we obtained differences in number of switches in the human sample that do not appear in the simulation with the LLM model.

Furthermore, LLM models are trained on written and spoken data (e.g., transcripts of people speaking, movies, YouTube videos). While human beings experience written and spoken language on their everyday lives, it is possible that the amount and quality of the information experienced between the humans we tested and the LLM model we used, differ to extents that may affect their performance in fluency tasks (Binz et al., 2025; Warstadt et al., 2023). For example, Dutch people are well known for the use of bikes. Consequently, when asked for bike parts, it does not seem that strange that our participants produced a number of words greater than for transportation means. This issue relates to the relative lack of “variability” of data that LLM models experience as well as how LLMs are designed to reproduce stable semantic relationships. Interestingly, a lack of variability in responses by LLM models has been attested in both category and letter fluency tasks (Qiu et al., 2025; Wang et al., 2025).

Finally, different LLM models (e.g., Llama, GPT) and parameterizations could have been used, changing the results (Liesenfeld et al., 2023). However, both the model and the parameters we used seem a good option, at least in the current time, to generate Dutch fluency data, mimicking what humans would do. Different from Qiu et al. (2025) we found no differences regarding word properties, while they did find that LLMs produced words that were in general less diverse than humans (e.g., more frequent, earlier age of acquisition, shorter). Further work seems needed, although a difference between Qiu et al. (2025) and our study is that we used category fluency tasks, while they administered letter fluency tasks. Future work could use a similar script to generate responses for more categories and subcategories (e.g., wild animals, pets, vegetables, dairy products, fruits from warm countries, public transport, car parts, sports with a ball, body parts, cities in the Netherlands, clothing, colors, countries, furniture, hobbies, musical instruments, natural phenomena, professions, school supplies, and sports) and/or compare different models to find appropriate benchmarks (e.g., Peeperkorn et al., 2024).

## Conclusion

Performance differs between category and subcategory fluency tasks. This finding may indicate differences in lexico-semantic search that occur when participants are asked to do these tasks. With the current paradigm we cannot discern that these differences may also be due to differences in the nature of category versus subcategory fluency tasks. Also, we cannot generalize our results to other types of tasks. Hence, the results ought to be replicated with other (more controlled) paradigms. The simulation with an LLM model seems relevant to provide novel insights into the study of fluency tasks—namely, possible differences as to how LLMs and humans may relate words, as well as other factors such as time pressure and task-order effects to which LLMs may be agnostic.

## Supplementary Information

Below is the link to the electronic supplementary material.

Supplementary file1 (DOCX 190 KB)

## Data Availability

The data and materials for all experiments are available at https://osf.io/gr6cd/

## Code availability

The code for all experiments is available at https://osf.io/gr6cd/

## Declarations

### Open practices statement

The data and materials for all experiments are available at https://osf.io/gr6cd/
