# Supplementary material for: Subcategory vs category fluency: Items and networks in healthy young adults and simulation with a large language model
Source: Mem Cognit. 2026 Apr 8;54(6):2270–88. doi: 10.3758/s13421-026-01869-3 (PMC13461686; doi:10.3758/s13421-026-01869-3)
Supplement: Supplementary file 1 [file 13421_2026_1869_MOESM1_ESM.docx]

**Subcategory vs category fluency: items and networks in healthy young adults and simulation with a large language model**

Adrià Rofes^1,2^*, Demi van Dijk^3^, Jeffrey C. Zemla^4^

^1^Center for Language and Cognition Groningen (CLCG), University of Groningen, The Netherlands

^2^Research School of Behavioural and Cognitive Neurosciences, University of Groningen. The Netherlands

^3^Koninklijke Kentalis, The Netherlands

^4^Department of Psychology, Syracuse University, USA

*Corresponding author:

Dr. Adrià Rofes. University of Groningen, Faculty of Arts - Neurolinguistics and Language Development, Oude Kijk in ‘t Jatstraat 26, 9712EK Groningen. The Netherlands. Telephone: [+31 50 36 38135](tel:+31503638135). E-mail: [a.rofes@rug.nl](mailto:a.rofes@rug.nl)

Running head: Subcategory fluency

Acknowledgements:

We thank Tommaso Caselli for useful discussions regarding the use of LLMs to extrapolate category and subcategory fluency data. AR received funding from the Dutch Research Council (NWO, 406.XS.01.050).

Open Practices Statement:

The data and materials for all experiments are available at https://osf.io/gr6cd/.

**Appendix A – Task instructions in English (translation) and original instructions in Dutch.**

English:

‘In this task you have to tell me as many different words as possible related to a given category. You can tell every word that pops into your mind, as long as it belongs to the given category. After one minute, I will stop the time and we will move on to the next category. There will be a total of 6 categories.’

‘We will start off with one practice category, to make sure that you know what to expect.’ ‘Let’s start.’

‘Tell me as many words as possible related to the category clothing.’ [it is not necessary to do this task for 1 minute. This is just to check that the participant understood the task].

‘Are you ready? Are there any more questions?’[pause]

‘Tell me as many words as possible related to the category: Animals, etc.

Stop after 1 minute.

Dutch:
*‘Bij deze test is het de bedoeling dat u in een minuut zo veel mogelijk woorden op noemt die u kunt bedenken bij een bepaalde categorie. U kunt ieder woord noemen dat in u opkomt. Na één minuut ga ik u vragen om te stoppen. Hierna gaan we verder met een andere categorie, er zijn in totaal 6 categorieën.’*

*‘Om te beginnen hebben we eerst een oefen categorie, om te kijken of u het goed begrijpt.’ ‘Daar gaan we nu mee beginnen.’*

*‘Noem zoveel mogelijk woorden die u kunt bedenken bij de categorie: kledingstukken’ [hoeft niet helemaal 1 minuut door te gaan, maar kijken of participant het principe snapt.]*

*‘Bent u er klaar voor of heeft u nog vragen?’ [pauze]*

*‘Noem zoveel mogelijk woorden als u kunt bedenken bij de categorie: Dieren, etc.*

*Stop na 1 minuut*

**Appendix B - Labels of schemes to measure cluster switches and average cluster size**

The animal fluency scheme is available here: <https://github.com/jmjvonk/2022_Rofes_SMART-MR>. The other fluency schemes are available for download here: <https://osf.io/gr6cd/>.

**Animal fluency**

African (e.g., monkey, antelope, buffalo), arachnid (e.g., spider, tick, tarantula), arctic (e.g., polar bear, narwal, walrus), Asian (e.g., panda, rhesus money, elephant), Australian (e.g., kangaroo, wallaby, koala), beasts of burden (e.g., lama, camel, horse, donkey), birds (e.g., albatross, condor, duck); bovine (e.g., cow, buffalo), canine (e.g., bulldog, boxer, golden retriever), dinosaur (e.g., velociraptor, brachiosaurus, triceratops), European (e.g., fox, wolf, wild pig), farm (e.g., goat, chicken, rabbit), feline (e.g., cat, jaguar, cheetah), fish (e.g., shark, dorado, guppy), forest (e.g., lynx, beer, deer), genus (mammal, marine fish, wild animal), insectivores (e.g., armadillo, mole, pangolin, bat), insects (e.g., ant, bee, mosquito), jungle (e.g., bonobo, tapir, toucan), North America (e.g., beaver, elk, nutria), pets (e.g., dog, gerbil, hamster), primates (e.g., gorilla, bonobo, monkey), reptiles (e.g., anaconda, cobra, gecko), rodents (e.g., rat, beaver, hedgehog), South America (e.g., quetzal, tamarin, jaguar), unicellular (e.g., ameba, bacteria, parasite), used for fur (e.g., lam, nutria, vicuna), water (e.g., alligator, ameba, dolphin), weasels (e.g., marmot, otter, skunk), worms (e.g., tapeworm, flatworm, earthworm), and zoo (e.g., gorilla, flamingo, giraffe).

**Farm animals**

Poultry and birds (e.g., chicken, turkey, duck, quail), dairy and meat (e.g., pigs, cows, goats, sheep, deer, fish), work (e.g., horses, oxen, mules), fur and fiber (e.g., mink, chinchilla, alpaca, llama, angora rabbit), and pets (e.g., guinea pigs, rabbits).

**Foods**

Fruits (e.g., apple, banana, pear), vegetables (e.g., carrots, lettuce, radish), grains (e.g., rice, oats, barley), protein (e.g., types of meat, poultry, fish, eggs, nuts, tofu), dairy (e.g., milk, yogurt, cheese, and plant-based alternatives), and fats and oils (e.g., olive oil, butter, salad dressings).

**Fruits**
Berries (e.g., strawberry, blueberry, raspberry, blackberry), citric (e.g., orange, lemon, lime, grape, tangerine), tropical (e.g., banana, mango, pineapple, papaya, kiwi), pome (e.g., apple, peer), stone (e.g., peaches, plums, cherries, apricots, nectarines), and melons (e.g., watermelon, cantaloupe, honeydew, muskmelon).

**Transport**

Land transport (e.g., bike, car, truck, bus, train), water transport (e.g., ship, boat, ferry, yacht, kayak, submarine), space/air transport (e.g., rocket, shuttle, probe, capsule), specialized transport (e.g., fire truck, ambulance, police car, tank, air jet, bulldozer, crane, excavator, dump truck).

**Bike parts**

Frame and form (e.g., mainframe, chassis, tubing, fork, saddle, fender, bottle rack), wheels and tires (e.g., wheel, rim, spoke, hub, tire), dive train (e.g., chain, crank, pedal, derailleur, cassette), brakes (e.g., break, lever, caliper, pad), handlebar and controls (e.g., lights, handlebar, stem, shifter, grip). We did not consider accessories (e.g., panniers, stickers, child seat).

**Appendix C – On-line closed card sorting experiment**

| **Task** | **Label** | **Score (card sorting)** | **Percentage of words** |
| --- | --- | --- | --- |
| Farm animals | Poultry & birds | 82.9 | 28.7 |
| Farm animals | Pets | 73.0 | 15.8 |
| Farm animals | Work | 60.4 | 13.9 |
| Farm animals | Dairy & meat | 60.2 | 32.7 |
| Farm animals | Fur & fiber | 48.5 | 8.9 |
| Foods | Fruits | 93.2 | 14.5 |
| Foods | Dairy | 89.0 | 16.1 |
| Foods | Vegetables | 82.0 | 27.4 |
| Foods | Protein | 80.4 | 19.4 |
| Foods | Grains | 68.0 | 13.7 |
| Foods | Fats & oils | 66.5 | 8.9 |
| Bike parts | Brakes | 98.9 | 13.6 |
| Bike parts | Wheels & tires | 85.3 | 22.0 |
| Bike parts | Drivetrain | 58.6 | 16.9 |
| Bike parts | Handlebar controls | 42.1 | 18.6 |
| Bike parts | Frame & form | 40.9 | 28.8 |
| Fruits | Melons | 87.3 | 8.1 |
| Fruits | Berries | 85.9 | 29.0 |
| Fruits | Citric | 80.8 | 21.0 |
| Fruits | Tropical | 69.9 | 21.0 |
| Fruits | Stone | 68.8 | 12.9 |
| Fruits | Pome | 65.5 | 8.1 |
| Transport | Water | 98.4 | 19.6 |
| Transport | Land | 83.6 | 63.7 |
| Transport | Space | 70.2 | 8.8 |
| Transport | Specialized | 64.5 | 7.8 |

Notes: Score (card sorting) = average score representing how often people chose the label, given all the words in each card sorting task.

**Appendix D – Code for LLM generated responses for replication and expansion**

#Code 1: Generated responses considering mean accuracy scores collected in human participants (replication)

import csv

from llama_cpp import Llama

\# Path to your GGUF model file

model_path = "./models/Mistral-Small-24B-Instruct-2501-Q4_K_M.gguf"

\# Initialize the model

llm = Llama(

model_path=model_path,

n_ctx=2048,

n_threads=8,

chat_format="llama-2",

)

\# Dutch instructions

instructions = (

"Bij deze test is het de bedoeling dat u in een minuut zo veel mogelijk woorden op noemt die u kunt bedenken bij een bepaalde categorie. "

"U kunt ieder woord noemen dat in u opkomt. Na één minuut ga ik u vragen om te stoppen. "

"Hierna gaan we verder met een andere categorie, er zijn in totaal 6 categorieën.\n"

)

\# Categories with approximate target word counts

categories = [

("Dieren", "animals", 25),

("Boerderijdieren", "farm animals", 13),

("Eten", "food", 23),

("Fruit", "fruit", 15),

("Vervoermiddelen", "means of transport", 15),

("Fietsonderdelen", "bicycle parts", 17),

]

num_participants = 50

rows = []

order = 1

for participant in range(1, num_participants + 1):

for dutch_cat, eng_cat, target in categories:

prompt = (

f"{instructions}"

f"Noem ongeveer {target} verschillende woorden bij de categorie: {dutch_cat}."

)

response = llm.create_chat_completion(

messages=[{"role": "user", "content": prompt}],

max_tokens=6000, # Adjust as needed to fit your expected word count

temperature=0.7, # To strike balance between creativity and conservative/repetitive answers

)

antwoord = response["choices"][0]["message"]["content"]

\# Split response into individual words (comma or newline separated)

words = [w.strip() for w in antwoord.replace('\n', ',').split(',') if w.strip()]

for word in words:

rows.append({

"Order": order,

"ID": participant,

"Task": eng_cat,

"Word": word

})

order += 1

csv_filename = "LLM_fluency_replication.csv"

with open(csv_filename, mode='w', newline='', encoding='utf-8') as file:

writer = csv.DictWriter(file, fieldnames=["Order", "ID", "Task", "Word"])

writer.writeheader()

for row in rows:

writer.writerow(row)

print(f"CSV file '{csv_filename}' created with {order-1} words.")

**Appendix E – Category and subcategory fluency generated by a Large Lange Model (LLM)**

**Methods**

**Pseudo-participants**
The data was generated by a LLM (see Methods). We prompted the model to generate responses for 50 pseudo-participants. We explicitly prompted the model to generate a similar number of words than in study 1 by considering mean accuracy scores previously collected in human participants.

**Materials and procedure**

We set up a local environment to run the Mistral-Small-24B-Instruct-2501-Dutch model in GGUF format using Python and the llama-cpp-python library (Mistral AI Team, 2025). We chose Mistral-Small-24B-Instruct-2501-Dutch because it combines state-of-the-art instruction-following and advanced reasoning capabilities with specific optimization for the Dutch language, making it highly suitable for study. This model is multilingual and open source, and efficient enough to run locally on consumer hardware, ensuring data privacy and low latency. Its fine-tuning on a large Dutch corpus ensures robust performance for Dutch prompts, while its 24 billion parameters deliver high-quality, knowledge-dense responses, all under a permissive Apache 2.0 license.

After installing the necessary tools, we downloaded the Dutch GGUF model from Hugging Face (huggingface.co). We then wrote two Python scripts to load the model, provide a Dutch prompt, and generate a text completion locally. The code was generated by Perplexity AI (Perplexity, 2025) and manually inspected and tested by one of the authors (AR). The script generated responses for the six fluency tasks used in this study (i.e., animals, farm animals, foods, fruit, transport, bicycle parts). For LLM generated words, we set the level of randomness of the responses (i.e., temperature) to 0.7 to avoid repetitive or overly conservative answers, in an attempt to capture what humans would do^[1]^.

With the same goal of replicating what humans do, we set the maximum number of tokens per response to 100. A token is a basic unit of text that the model processes, which can be a whole word, part of word, or individual characters. Again, there is no standard to decide the number of tokens when generating fluency data. The scripts we used to generate the LLM datasets can be found in a repository (<https://osf.io/gr6cd/>.).

**Scoring**
Once the data was generated, we followed the same procedures as in Study 1 for tallying correct words, number of cluster switches and average cluster size, word properties, subcategory responses within category fluency, and generation of word networks. Some extra manual preprocessing of the LLM generated data was needed to remove foreign content to the task, such as “comments” made by the LLM, extra spaces and/or stops, numbers or hyphens (e.g., 1. Dog, 2. Kat; - dog, - kat). AR and DvD were responsible for these steps.

To minimize the number of empty values, we searched for alternative words, same as in Study 1. When this was not possible, we imputed the value (mean score per pseudo-participant for each word property). The number and percentage of imputed values for each property were: frequency (86/3274, 2.7% missing values), orthographic neighborhood (295/3274; 9.9% missing values), phonological neighborhood (295/3274; 9.9% missing values), word length in graphemes (0% missing values).

**Analyses**

The statistical analyses are the same than in Study 1.

**Results**

*Differences between category and subcategory fluency*

The raw data and scripts can be accessed here https://osf.io/gr6cd/. Table A2 includes descriptive statistics for the variables group (categories, subcategories) and for each task. Table A3 includes descriptive statistics for network metrics. The comparisons can be checked in Table A4. Two aspects of the LLM generated data are worth noting: we found 85 foreign words amounting to 2.5% of the generated sample.

*Correct words*

We found an interaction between *group* and *field* (χ²(2) = 14.50, p < .001). Pseudo-participants produced more correct words in the category task *animal fluency* (mean = 13, SD = 2) than in the subcategory task *farm animals* (mean = 12, SD = 2) (χ²(1) = 5.25, p = .022). They also produced more words in the category task *transport* (mean = 13, SD = 1) than the subcategory task *bike parts* (mean = 9, SD = 2) (χ²(1) = 27.43, p < .001). No differences were detected between the category task *foods* (mean = 11, SD = 3) and the subcategory task *fruit* (mean = 11, SD = 2) (χ²(1) = 0.0008, p = .92).

*Number of cluster switches and average cluster size*

We found interactions between *group* and *field* for both number of cluster switches (χ²(2) = 233.25, p < .001) and average cluster size (χ²(2) = 48.46, p < .001). Further analyses revealed that pseudo-participants produced fewer switches in the category fluency tasks *foods* (mean = 1.14, SD = 1.1) compared to the subcategory fluency tasks *fruits* (mean = 8.56, SD = 2.36) (χ²(1) = 9.173, p < .001). We also found differences regarding switches between the category fluency task *animals* (mean = 6.02, SD = 1.94) and the subcategory fluency task *farm animals* (mean = 7.65, SD = 1.25) (χ²(1) = 312.2, p < .001) and between the category fluency *transport* (mean = 5.85, SD = .91) and the subcategory *bike parts* (mean = 4.73, SD = 1.39) (χ²(1) = 5.443, p = .019).

Regarding cluster size, pseudo-participants produced bigger clusters for the category fluency tasks *animals* (mean = 2, SD= .6), *foods* (mean = 1.27, SD=.4), and *transport* (mean = 1.41, SD = .16), than for the subcategory fluency tasks *farm animals* (mean = 1.28, SD = .12) (χ²(1) = 106.24, p < .001), *fruits* (mean = 1.14 SD = .16) (χ²(1) = 4.869, p = .027), and *bike parts* (mean = 1.22, SD = .21) (χ²(1) = 30.74, p < .001).

*Word properties*

We found interactions between *group* and *field* for all word properties (see Table A4). Relative to word frequency, pseudo-participants produced more frequent words for the category fluency tasks *foods* (mean = 2.73, SD = .11) and *transport* (mean = 3, SD = .14) than for the subcategory fluency tasks *fruits* (mean = 1.96, SD = .9) (χ²(1) = 272.57, p < .001) and *bike parts* (mean = 2.16, SD = .19) (χ²(1) = 180.86, p < .001) respectively. Pseudo-participants produced more frequent words in the subcategory task *farm animals* (mean = 2.92, SD = .21) than in the category task *animals* (mean = 2.82, SD= .17) (χ²(1) = 15.205, p < .001).

Regarding orthographic neighborhood and phonological neighborhood pseudo-participants produced words with fewer orthographic/phonological neighbors for the category fluency tasks *animals* and *transport* than for to the subcategory fluency tasks *farm animals* and *bike parts*, respectively (Table 3, Table A2). However, pseudo-participants produced words in the category fluency *foods* that had more orthographic/phonological neighbors than the subcategory *fruits* (Table 3, Table A2).

Regarding word length, pseudo-participants produced longer words for the subcategory tasks *fruits* (mean = 6.10, SD = .33) and *bike parts* (mean = 6.66, SD = .67) compared to the category tasks *foods* (mean = 5.50, SD = .49, χ²(1) = 48.444, p < .001) and transport (mean = 6.12, SD = .37, χ²(1) = 30.703, p < .001). However, pseudo-participants produced longer words in the category fluency task *animals* (mean = 5.29, SD = .5) than in the subcategory fluency task *farm animals* (mean = 4.58, SD = .51, χ²(1) = 57.084, p < .001). See Figure 2.

*Word network metrics*

Table A3 includes descriptive statistics for network metrics. From a descriptive standpoint, *category fluency* and *subcategory fluency* has nearly identical clustering coefficients and density. *Category fluency* had lower average node degree, and greater number of edges, number of nodes, average shortest-path length (ASPL) and diameter. However, these patterns were inconsistent across pairs (i.e., *animals* versus *farm animals*, *foods* versus fruits, and *transport* versus *bike parts*).

*Subcategory responses in each corresponding category fluency*

Pseudo-participants listed more farm animals in *farm animal fluency* (mean =11.53, SD = 1.5) than *animal fluency* (mean = 7.74, SD = 2.07, *t*(80.234) = 9.963, *p* < .001). They also listed more fruits in *fruits fluency* (mean = 10.86, SD = 2.63) than *foods fluency* (mean = 0.25, SD = .534, *t*(46.515) = 26.16, *p* < .001). Average farm animal cluster size was not different in both *farm animal fluency* (mean = 1.27, SD = .12) and *animal fluency* (mean = 1.27, SD = .18, *t*(79.41) = -0.063, *p* = .94). The comparison between for average fruit cluster size could not be made due to the lack of variability in *foods fluency* (mean = 1, SD = .0) compared to *fruit fluency* (mean = 1.14, SD = .12) than *foods fluency*.

Table A1 Comparisons between and within tasks: correct words, word properties, cluster size and number of cluster switches (Human data)

|  | **Corr.words** | **Frequency** | **Orth.Neigh** | **Phon.Neigh** | **Length** | **Cluster Size** | **N.Switches** |
| --- | --- | --- | --- | --- | --- | --- | --- |
| **Group*field** | χ²(2) = 124.67, p < .001* | χ²(2) = 227.12, p < .001* | *F*(2, 235) =  75.647,  *p* < .001* | *F*(2, 235) =  93.029,  *p* < .001* | *F*(2, 235) =  32.702,  *p* < .001* | χ²(2) = 168.83, p < .001* | *F*(2, 235) =  80.745,  *p* < .001* |
| **Animals vs farm animals** | χ²(1) = 181.48, p < .001* | χ²(1) = 71.498, p < .001* | *F*(1, 47) =  147.86,  *p* < .001* | *F*(1, 47) =  139.21,  *p* < .001* | *F*(1, 47) =  19.463,  *p* < .001* | χ²(1) = 79.291, p < .001* | *F*(1, 47) =  17.669,  *p* < .001* |
| **Foods vs fruits** | χ²(1) = 70.969, p < .001* | χ²(1) = 83.432, p < .001* | *F*(1, 47) =  29.149  *p* < .001* | *F*(1, 47) =  85.279,  *p* < .001* | *F*(1, 47) =  33.445  *p* < .001* | χ²(1) = 22.872, p < .001* | *F*(1, 47) =  168.91,  *p* < .001* |
| **Transport vs bike parts** | χ²(1) = 6.299, p = .001* | χ²(1) = 132.38, p < .001* | *F*(1, 47) =  20.836,  *p* < .001* | *F*(1, 47) =  17.455,  *p* < .001* | *F*(1, 47) =  29.006,  *p* < .001* | χ²(1) = 112.02, p < .001* | *F*(1, 47) =  101.62,  *p* < .001* |

Notes: Corr.Words = Number of correct words; Orth.Neigh = Orthographic neighborhood; Phon.Neigh = Phonological neighborhood, Length = Length in graphemes; Cluster.Size = Average cluster size (static); N.Switches = Number of cluster switches (static).

Table A2 Descriptive statistics per task group (category, subcategory), and per individual task for correct words, word properties, average cluster size and number of cluster switches. LLM-generated data (study 2).

| **Task** | **Corr.Words** | **Frequency** | **Phon.Neigh** | **Orth.Neigh** | **Length** | **N.switches** | **Cluster size** |
| --- | --- | --- | --- | --- | --- | --- | --- |
| **Category** | 12 (2) | 2.85 (.18) | 8.83 (2.56) | 6.05 (1.95) | 5.63 (.58) | 4.31 (2.67) | 1.56 (1.21) |
| **Subcategory** | 11 (2) | 2.34 (.44) | 8.02 (4.82) | 5.99 (3.64) | 5.78 (1.02) | 7.02 (2.38) | 1.21 (.16) |
| **Animals** | 13 (2) | 2.82 (.17) | 10.28 (2.28) | 7.83 (1.69) | 5.29 (.5) | 6.02 (1.94) | 2.00 (.6) |
| **Farm animals** | 12 (2) | 2.92 (.13) | 13.96 (1.92) | 10.57 (1.44) | 4.58 (.51) | 7.65 (1.25) | 1.28 (.12) |
| **Foods** | 11 (3) | 2.73 (.11) | 9.50 (2.57) | 5.87 (1.57) | 5.50 (.49) | 1.14 (1.1) | 1.27 (.41) |
| **Fruit** | 11 (2) | 1.96 (.09) | 3.00 (1.05) | 2.38 (1.03) | 6.10 (.33) | 8.56 (2.36) | 1.14 (.12) |
| **Transport** | 13 (1) | 3.00 (.14) | 6.66 (.82) | 4.42 (.46) | 6.12 (.37) | 5.85 (.91) | 1.41 (.16) |
| **Bike parts** | 9 (2) | 2.16 (.19) | 7.29 (1.72) | 5.17 (1.19) | 6.66 (.67) | 4.73 (1.39) | 1.22 (.21) |

Notes: Corr.Words = Number of correct words; Phon.Neigh = Phonological neighborhood, Orth.Neigh = Orthographic neighborhood;

Length = Length in graphemes; Cluster Size = Average cluster size (static); N.Switches = Number of cluster switches (static).

Table A3 Word network metrics. LLM-generated data (study 2).

| **Task** | **Clustering coefficient** | **Density** | **Number edges** | **Number nodes** | **Average node degree** | **ASPL** | **Diameter** |
| --- | --- | --- | --- | --- | --- | --- | --- |
| **Category** | 0.271 | 0.028 | 227 | 128 | 4.072 | 5.032 | 10 |
| **Subcategory** | 0.273 | 0.028 | 197 | 120 | 4.845 | 4.668 | 12 |
| **Animals** | 0.189 | 0.027 | 39 | 54 | 4.286 | 1.727 | 9 |
| **Farm animals** | 0.183 | 0.045 | 21 | 31 | 2.818 | 1.532 | 7 |
| **Foods** | 0.177 | 0.024 | 26 | 47 | 2.556 | 1.273 | 5 |
| **Fruits** | 0.054 | 0.030 | 15 | 32 | 2.628 | 1.395 | 6 |
| **Transport** | 0.136 | 0.023 | 26 | 48 | 3.255 | 1.445 | 7 |
| **Bike parts** | 0.111 | 0.015 | 13 | 42 | 2.444 | 0.651 | 4 |

Notes: ASPL = Average short path length.

Table A4 Comparisons between and within tasks: correct words, word properties, cluster size and number of cluster switches (LLM data)

|  | **Corr.words** | **Frequency** | **Orth.Neigh** | **Phon.Neigh** | **Length** | **Cluster Size** | **N.Switches** |
| --- | --- | --- | --- | --- | --- | --- | --- |
| **Group*field** | χ²(2) = 14.50, p < .001* | χ²(2) = 344.96,  p < .001* | χ²(2) = 192.55,  p < .001* | χ²(2) = 290.87, p < .001* | χ²(2) = 106.83, p < .001* | χ²(2) = 233.25, p < .001* | χ²(2) = 48.46, p < .001* |
| **Animals vs farm animals** | χ²(1) = 5.25, p = .022* | χ²(1) = 15.205, p < .001* | χ²(1) = 76.605  , p < .001* | χ²(1) = 82.772, p < .001* | χ²(1) = 57.084, p < .001* | χ²(1) = 106.24, p < .001*), | χ²(1) = 312.2, p < .001 |
| **Foods vs fruits** | χ²(1) = 27.43, p < .001* | χ²(1) = 272.57, p < .001* | χ²(1) = 85.389, p < .001* | χ²(1) = 145.98, p < .001* | χ²(1) = 48.444, p < .001* | χ²(1) = 4.869, p = .027* | χ²(1) = 9.173, p < .001* |
| **Transport vs bike parts** | χ²(1) = 0.0008, p = .92 | χ²(1) = 180.86, p < .001* | χ²(1) = 18.125, p < .001* | χ²(1) = 5.191, p < .001* | χ²(1) = 30.703, p < .001* | χ²(1) = 30.74, p < .001* | χ²(1) = 5.443, p = .019 |

Notes: Corr.Words = Number of correct words; Orth.Neigh = Orthographic neighborhood; Phon.Neigh = Phonological neighborhood, Length = Length in graphemes; Cluster.Size = Average cluster size (static); N.Switches = Number of cluster switches (static).

Table A5 Lexical diversity measures for human data and LLM data (all tasks considered)

| **Metric** | **Human data** | **LLM data** |
| --- | --- | --- |
| **Total Tokens** | 5,355 | 3,813 |
| **Total Types** | 1,100 | 570 |
| **% Hapax Types** | 49.55% | 54.39% |
| **Type–Token Ratio (TTR)** | 0.205 | 0.149 |
| **Mean TTR (sliding window 15 words)** | 0.9988 | 0.9907 |
| **Mean TTR (sliding window 100 words)** | 0.9240 | 0.8313 |
| Notes: Hapax indicates types that appear “only once” in each entire dataset. | | |

Figure A1 Correlations between Human and LLM-data (all tasks aggregated)

Note: Red Dotted Line (Identity Line): Represents the line y=xy = xy=x, indicating where human and LLM values would be equal. Blue Line (Regression Line): Represents the best-fit line for the relationship between human and LLM values, showing the trend in the data. The high correlation patterns indicate similar results between the human and LLM values. Visual inspection of the patterns showed more similar results for Mean Phonological Neighborhood and Mean Orthographic Neighborhood than for Mean Frequency and Mean Length in Graphemes.

1. To the best of our knowledge, higher temperature leads to similar results to those of humans, at least in letter fluency (Qiu et al., 2025). However, there is no set standard regarding for the temperature parameter in any fluency task. We set out for 0.7 after running comparisons between total word count and word frequency between LLM datasets generated with different temperature parameters. Also, we performed a qualitative analysis of the responses: For word count, a generalized linear mixed-effects model (glmer) with a Poisson distribution and random intercepts for pseudo-participant and task showed that model-adjusted means (± SE, on the log scale) were 2.56 ± 0.02 for 0.7 temperature, 2.60 ± 0.02 for 0 temperature, and 2.63 ± 0.02 for 1 temperature. Pairwise comparisons (Tukey-adjusted) indicated that 1 temperature was significantly higher than 0.7 temperature (p = .013), while differences between 0.7 and 0 temperature (p = .21) and between 0 and 1 temperature (p = .48) were not significant. For word frequency, a linear mixed-effects model (lmer) with random intercepts for pseudo-participant and task showed that model-adjusted means (± SE) for mean frequency were 2.63 ± 0.16 for 0.7 temperature, 2.59 ± 0.16 for 1 temperature, and 2.55 ± 0.16 for 0 temperature. Pairwise comparisons (Tukey-adjusted) indicated that both 0.7 temperature (p < .001) and 1 temperature (p = .035) were significantly higher than 0 temperature, while 0.7 and 1 temperature did not differ significantly (p = .13). The qualitative analysis showed that the responses for temperature included little lexical and morphological variation. For example, for the word kip (chicken) we only obtained “kip”. However, for temperatures 0.7 and 1 we obtained, kippen (chickens), kippetje (little chicken), kippie (little chicken, another variation). Interestingly, no matter what temperature we used we obtained most words in Dutch, but also words in languages such as English (e.g., melon, soup) German (e.g., Schmetterling, Schwein, butterfly, pig) and Spanish (e.g., pera, pear). When the LLM produced “comments” (e.g., “Natuurlijk” – of course; “Ik begin met de categorie “Dieren.” Ik zal zo veel mogelijk woorden noemen in de komende minuut. – I’ll start with the category “Animals.” I’ll name as many words as I can in the next minute.), we saw more variation when the temperature was 0.7 or 1 vs 0.
